# Reduction in the olfactory ability in aging *Mitf* mutant mice without evidence of neurodegeneration

**DOI:** 10.3389/fragi.2024.1462900

**Published:** 2024-10-25

**Authors:** Fatich Mechmet, Eiríkur Steingrímsson, Petur Henry Petersen

**Affiliations:** ^1^ Department of Anatomy, Biomedical Center, Faculty of Medicine, University of Iceland, Reykjavik, Iceland; ^2^ Department of Biochemistry and Molecular Biology, Biomedical Center, Faculty of Medicine, University of Iceland, Reykjavik, Iceland

**Keywords:** age-related decline, olfactory bulb, olfactory function, *Mitf* mutation, neuronal hyperactivity, potassium channels

## Abstract

Age-related decline occurs in most brain structures and sensory systems. An illustrative case is olfaction. The olfactory bulb (OB) undergoes deterioration with age, resulting in reduced olfactory ability. A decline in olfaction is also associated with early symptoms of neurodegenerative diseases, including Alzheimer’s disease (AD) and Parkinson’s disease (PD). However, the underlying reasons are unclear. The microphthalmia-associated transcription factor (MITF) is expressed in the projection neurons (PNs) of the OB–the mitral and tufted (M/T) cells. Primary M/T cells from *Mitf* mutant mice show hyperactivity, potentially attributed to the reduced expression of a key potassium channel subunit, *Kcnd3*/Kv4.3. This influences intrinsic plasticity, an essential mechanism involving the non-synaptic regulation of neuronal activity. As neuronal hyperactivity often precedes neurodegenerative conditions, the current study aimed to determine whether the absence of *Mitf* causes degenerative effects during aging. Aged *Mitf* mutant mice showed reduced olfactory ability without inflammation. However, an increase in the expression of potassium channel subunit genes in the OBs of aged *Mitf* ^*mi-vga9/mi-vga9*^ mice suggests that during aging, compensatory mechanisms lead to stabilization.

## Introduction

Aging is characterized by progressive deterioration. This includes loss of cognitive functions and reduction in the efficiency of sensory systems. Loss of sensory ability can also be a part of neuronal pathologies. The olfactory ability, the detection and discrimination of odors, shows an age-dependent decrease in humans (Rawson et al., 2012; Schubert et al., 2017) and rodents (Patel and Larson, 2009). However, olfactory impairment is also associated with a wide range of neurodegenerative and cognitive diseases, including Alzheimer’s disease (AD) (Murphy, 2019; Tzeng et al., 2021) and Parkinson’s disease (PD) (Doty, 2012; Fullard et al., 2017). Moreover, partial or total loss of olfactory ability (hyposmia or anosmia) contributes to depression (Sabiniewicz et al., 2022) and affects daily life (Schäfer et al., 2021). Olfactory dysfunction is therefore both a possible biomarker and a causal factor of disease. It is therefore important to investigate the underlying pathological mechanisms of olfactory dysfunction during aging.

The olfactory bulb (OB) is the first structure in the central nervous system (CNS) responsible for processing olfactory information. It is a well-defined and multi-layered structure with well-characterized neuronal subtypes (Nagayama et al., 2014). The olfactory process starts at the olfactory sensory neurons (OSNs) in the olfactory epithelium (OE), which detect odor molecules and synapse with two kinds of projection neurons (PNs), the mitral and tufted (M/T) cells and periglomerular cells (PGCs) in the OB (Lodovichi and Belluscio, 2012). The M/T cells transmit information to the piriform cortex (PCx) and other olfactory-associated brain areas, such as the olfactory tubercle (OT), amygdala, and orbitofrontal cortex (OCx) (Mori et al., 2013). Any changes in neuronal turnover, function, or morphology can affect the OB circuitry, which may contribute to impaired olfaction. The known impairments that cause an age-related decline in the olfactory system include structural, molecular, and functional modulations in the OE, main olfactory bulb (MOB), and other regions that are involved in olfactory processing (Doty and Kamath, 2014).

The microphthalmia-associated transcription factor (*Mitf)* encodes a member of the *Myc* supergene family of basic helix–loop–helix zipper (bHLH-Zip) transcription factors (Goding and Arnheiter, 2019; Steingrímsson et al., 2004). The MITF protein, which is known as a master regulator of melanocytes, also plays central roles in mast cells (Oppezzo and Rosselli, 2021) and osteoclasts (Lu et al., 2010; Steingrimsson et al., 2002) and has a distinct expression in the M/T cells (Atacho et al., 2020; Ohba et al., 2015). Previous studies have shown that *Mitf* plays a role in the intrinsic plasticity of primary M/T cells of the mouse OB. Its loss leads to increased neuronal excitability (Atacho et al., 2020). This suggests a role in homeostatic plasticity, which refers to the adjustment of neuronal activity towards a set point (Turrigiano, 2012). Primary PNs from young *Mitf* ^*mi-vga9/mi-vga9*^ mice are hyperactive, likely due to a reduced expression of potassium channel subunits (Atacho et al., 2020). As neuronal hyperactivity is a hallmark feature of the early phase of neurodegeneration (Chase and Markopoulou, 2020; Hector and Brouillette, 2020; Marin et al., 2018; Targa Dias Anastacio et al., 2022), it is of interest to determine whether the lack of *Mitf* has detrimental effects in the aged mouse OB. This question was addressed by examining markers of inflammation, evaluating the number of PNs, and analyzing global gene expression. The results suggest a buffering of neuronal activity via compensatory mechanisms in aging *Mitf* ^*mi-vga9/mi-vga9*^ mice. The study contributes to further identifying and understanding the mechanisms behind CNS adaptation to neuronal activity dysregulation during aging.

## Material and methods

### Animals

All *in vivo* procedures were approved by the Committee on Experimental Animals and were in accordance with Regulation 460/2017 and European Union Directive 2010/63 (license number 2013-03-01). Wild-type (C57BL/6J) and homo- and heterozygous *Mitf* ^*mi-vga9*^ mutant mice were used in this study. The *Mitf* ^*mi-vga9*^ mutation is a loss-of-function mutation caused by transgene insertion affecting the expression of *Mitf* (Hodgkinson et al., 1993; Tachibana et al., 1992). Mice were housed at the mouse facility at the University of Iceland, in groups of two to three per cage under controlled conditions (21°C–22°C; 12 h light/12 h dark). Unless indicated otherwise, food and water were provided *ad libitum.*

### Behavior: hidden food assay

The hidden food assay was performed during the active phase of mice in the dark cycle. Three age groups of mice, namely, 1–2 months old (five female and five male C57BL/6J mice; four female and six male *Mitf* ^*mi-vga9/+*^ mice; and five female and five male *Mitf* ^*mi-vga9/mi-vga9*^ mice); 8–12 months old (six female and four male C57BL/6J mice; one female and nine male *Mitf* ^*mi-vga9/+*^ mice; and five female and five male *Mitf* ^*mi-vga9/mi-vga9*^ mice); and 14–27 months old (six female and four male C57BL/6J mice; five female and five male *Mitf* ^*mi-vga9/+*^ mice; and six female and three male *Mitf* ^*mi-vga9/mi-vga9*^ mice), were used in the assay. The mice were first habituated to the test environment by placing them individually in cages for 24 h. Food and water were provided *ad libitum,* and cocoa puffs (Nesquik General Mills) were placed in each cage in a small Petri dish (four pieces per cage) for 12 h. The consumption of cereals was monitored for 2 consecutive days to ensure that the cereals were palatable. Following overnight (O/N) starvation (experimental license number 2016-05-01), the mice were kept in an odor-free room for 1 h with no water, food, or cereals. Each mouse was tested separately. In the assay, a cereal was hidden in an opposite corner under the bedding in a new cage, and the time spent finding it was measured.

### Immunofluorescence

The mice were transcardially perfused (license number: 2014-07-02) with 1 × phosphate-buffered saline (PBS; Gibco, cat# 18912-014), followed by 4% paraformaldehyde (4% PFA; Sigma-Aldrich, cat# P6148) in 1 × PBS, pH 7.4. Subsequently, brains were post-fixed with 4% PFA in 1 × PBS at 4°C O/N. Following this, the brains were rinsed with 1 × PBS for 2 days. OBs from young and aged C57BL/6J and *Mitf* ^*mi-vga9/mi-vga9*^ mice were dissected and placed in a Peel-A-Way disposable plastic tissue Embedding Molds (Polysciences, Inc.) filled with 5% agarose (Invitrogen, cat# 15517-014) dissolved in dH_2_O. The OBs were sectioned into 50 μm thin sections at room temperature (RT) using a microtome, Microm HM 650V (Thermo Scientific), and were maintained at 4°C until further use. The sections were blocked in blocking buffer composed of 10% normal goat serum (NGS; Gibco, cat# 16210-064) and 0.3% Triton X-100 (Sigma, cat# T8787) in 1 × PBS for 1 h at RT and were then incubated with primary antibodies diluted in the blocking buffer at 4°C O/N. The primary antibodies used in this study were as follows: rabbit polyclonal anti-GFAP (1:1,000; Abcam, ab7260), rabbit monoclonal [EPR16588] anti-Iba-1 (1:1,000; Abcam, ab178846), rabbit monoclonal [EPR21950] anti-Tbr2/Eomes (1:1,000; ab216870), and rabbit monoclonal [EPR8138(2)] anti-Tbr1 (1:1,000; ab183032). Following three washing steps with 1 × PBS for 5 min each, the sections were incubated with secondary antibodies, Alexa Flour 546 IgG anti-rabbit for anti-GFAP and anti-Iba-1, Alexa Flour goat 647 IgG anti-rabbit for anti-Tbr2/Eomes and anti-Tbr1 (Life Technologies; each diluted at 1:1,000 in the blocking buffer), and DAPI (1:1,000; Sigma, cat# D9542) for 1 h at RT in the dark. After washing with 1 × PBS, the tissues were placed on Menzel–Gläser Superfrost Plus microscope slides (Thermo Scientific, cat# 2621573) and mounted using Fluoromount Aqueous Mounting Medium (Sigma-Aldrich, cat# F4680). Imaging was performed at 20 × magnification using 20 confocal Z-stacks. Four images were obtained from each section, two from lateral and two from the medial OB. The sex of the animals used for staining of each antibody was as follows: for GFAP and Iba-1, young mice included six female and four male C57BL/6J and three female and seven male *Mitf* ^*mi-vga9/mi-vga9*^, while aged mice included five female and five male C57BL/6J and six female and four male *Mitf* ^*mi-vga9/mi-vga9*^. For Tbr2/Eomes staining, two female and three male C57BL/6J and four female and two male *Mitf* ^*mi-vga9/mi-vga9*^ mice were used. For Tbr1 staining, young mice included two female and one male C57BL/6J, and three male *Mitf* ^*mi-vga9/mi-vga9*^, while aged mice included one female and two male C57BL/6J and two female and one male *Mitf* ^*mi-vga9/mi-vga9*^ mouse.

### Golgi–Cox staining for light microscopy

Golgi–Cox staining was performed according to the protocol described by Vints et al. (2019). Briefly, PFA-fixed OB sections from aged C57BL/6J and *Mitf* ^*mi-vga9/mi-vga9*^ (four male C57BL/6J; two female and two male *Mitf* ^*mi-vga9/mi-vga9*^) mice were incubated in Golgi–Cox solution, a mixture of 5% mercury chloride (Sigma-Aldrich, cat# M1136), 5% potassium dichromate (Sigma-Aldrich, cat# 207802), and 5% potassium chromate (Sigma-Aldrich, cat# 216615), for 15 days at RT in the dark. On day 15, the sections were washed three times for 5 min with dH_2_O and transferred to 28% ammonium hydroxide (Sigma-Aldrich, cat# 221228) in dH_2_O for 30 min at RT with rotation. Following three washing steps with dH_2_O, the sections were incubated in 15% ILFORD RAPID FIXER (Harman Technology Limited) in dH_2_O for 10 min at RT and then washed three times with dH_2_O. The sections were placed on Menzel–Gläser Superfrost Plus slides (Thermo Scientific, cat# 2621573), dried at RT, and mounted with the Mowiol 4-88 (Aldrich, cat# 81381) mounting medium. Slides were imaged using a Leica light microscope.

### Western blotting

Mice were sacrificed by cervical dislocation. OBs from young and aged C57BL/6J and *Mitf* ^*mi-vga9/mi-vga9*^ (young mice: one female and two male C57BL/6J, two female and one male *Mitf* ^*mi-vga9/mi-vga9*^; aged mice: two female and one male C57BL/6J, one female and two male *Mitf* ^*mi-vga9/mi-vga9*^) mice were weighed and homogenized in radioimmunoprecipitation assay (RIPA) buffer (250 mM NaCl, 1% IGEPAL CA-630, 0.5% sodium deoxycholate, 0.1% sodium dodecyl sulfate, and 50 mM Tris-HCl, pH 8.0) containing 1 × Halt protease and phosphatase inhibitor cocktail (Thermo Scientific, cat# 78440). Lysates were spun at 16,000 × g for 10 min at 4°C, and the supernatant was transferred to a new tube. After determining the protein concentration with a Bradford protein assay using the Bradford reagent (Sigma-Aldrich, cat# B6916), each sample was diluted with an appropriate volume of RIPA buffer mixed with 1 × Halt protease and phosphatase inhibitor cocktail to achieve a final amount of 20 µg protein. Following this, 2 × sample buffer (4% sodium dodecyl sulfate, 20% glycerol, bromophenol blue 0.02%, and 120 mM Tris-HCl, pH 6.8) containing 5% β-mercaptoethanol (Sigma-Aldrich, cat# M6250) was added to each sample at a 1:1 ratio, and the lysates were heated at 95°C for 5 min. The protein lysates were run on 12.5% SDS-PAGE gel and transferred onto a 0.2-µm PVDF membrane (Thermo Scientific, cat# 88520). The membrane was incubated in a blocking buffer consisting of 5% non-fat dried milk in 1 × Tris-buffered saline/0.1% Tween 20 (TBS-T) for 1 h at RT. Following this, the membranes were incubated O/N at 4°C with the following primary antibodies diluted in the blocking solution: rabbit polyclonal anti-GFAP (1:1,000; Abcam, cat# ab7260), rabbit monoclonal [EPR16588] anti-Iba-1 [1:1,000; Abcam, ab178846 (EPR16588)], and mouse monoclonal anti-beta actin (1:5000; ab8224) or mouse monoclonal anti-GAPDH (1:5,000; ab8245). The membranes were washed with 1 × TBS-T and incubated for 1 h at RT with the following secondary antibodies diluted at 1:10,000 in the blocking buffer: IRDye goat anti-rabbit IgG 800 (green; LI-COR Biosciences) or IRDye donkey anti-mouse IgG1 680 (red; LI-COR Biosciences). After washing with 1 × TBS-T, the membranes were imaged using the Odyssey CLx Imager (LI-COR Biosciences).

### RNA sequencing and data analysis

OBs from aged C57BL/6J and *Mitf* ^*mi-vga9/mi-vga9*^ mice (two female and one male C57BL/6J; two female and one male *Mitf* ^*mi-vga9/mi-vga9*^) were dissected and flash-frozen in liquid nitrogen. Tissues were homogenized in 1 × DNA/RNA protection reagent (New England Biolabs Inc., Part: T2011-1), and RNA was isolated using a commercially available kit (Monarch Total RNA Miniprep Kit; New England Biolabs Inc., cat# T2010S).

The quality (RIN score) and quantity of the isolated total RNA samples were evaluated using the DNA 5K/RNA chip on the LabChip GX instrument. To create cDNA libraries from poly-A mRNA, Illumina’s TruSeq RNA v2 Sample Prep Kit (Illumina, RS-122-2001) was used. This process involved isolating poly-A mRNA from the total RNA samples (0.2–1 μg input) through hybridization with poly-T beads.

Subsequently, the poly-A mRNA was fragmented at 94°C with divalent cations, followed by initiating first-strand cDNA synthesis using random hexamers and SuperScript IV Reverse Transcriptase (Invitrogen, cat# 18090010). Following this, second-strand cDNA synthesis, unique dual-indexed adapter ligation, and PCR amplification were performed. The resultant cDNA sequencing libraries were evaluated using the LabChip GX system and then diluted to 3 nM and stored at −20°C.

For the sequencing, the samples were pooled, and clustering was carried out on NovaSeq S4 flow cells. The sequencing approach involved paired-end sequencing using the XP workflow on NovaSeq 6000 instruments (Illumina). Basecalling was performed in real-time using RTA v3.4.4. The process of demultiplexing BCL files and generating FASTQ files was done using bcl2fastq2 v.2.20.

A companion package of Kallisto (v 0.46.1) (Bray et al., 2016) was used to quantify transcript abundance following the pseudoalignment of RNA-seq data to the mouse reference genome (*Mus musculus*.GRCm38.96) (Yates et al., 2016). Differentially expressed (DE) genes in the OBs of aged C57BL/6J and *Mitf* ^*mi-vga9/mi-vga9*^ mice (n = 3 per genotype) were identified using Sleuth (Pimentel et al., 2017). The significance of DE genes (p-/q-values) and the fold change (beta estimate) were determined using the likelihood test (LRT) intersected in Sleuth.

Volcano plots were used to represent DE genes between aged *Mitf* ^*mi-vga9/mi-vga9*^ and C57BL/6J OBs by plotting the significance on the *y*-axis and cut-off of fold change on the *x*-axis (q-value ≤ 0.05; cut-off of |b-value (fold change)| ≥ 0.7) using R packages *“ggplot2”* and *“ggrepel*.*”* R package *“cowplot”* was used to create bar graphs showing TPM values of each gene between aged *Mitf* ^*mi-vga9/mi-vga9*^ and C57BL/6J. Functional enrichment analyses (GO terms) in molecular functions (MF) and enrichment analysis of pathways (KEGG) were generated combining *p*-value ≤ 0.05 and cut-off of |b-value (fold change)| ≥0.7 with *“clusterProfiler”* in the Bioconductor R package (Yu et al., 2012).

### Quantification of Tbr2/Eomes and Tbr1-positive cells

Tbr2/Eomes and Tbr1-positive cells from OB sections of young and aged C57BL/6J and *Mitf* ^*mi-vga9/mi-vga9*^ mice were counted using ImageJ software. Briefly, the software application was uploaded with oib images of the lateral and medial OB, showing the GL, EPL, and MCL. After stacking the image at *“Maximum Intensity”* using the *“Z-project”* analyzing method of the software application, the image channels were split. Subsequently, the channel showing Tbr2/Eomes or Tbr1 antibody staining was chosen. To make weaker stained cells more distinguishable, the channel was converted to grayscale, and Tbr2/Eomes and Tbr1-positive cells in the GL, EPL, and MCL were counted manually in the lateral and medial OB. The average number of cells per image was calculated.

### Analysis of Golgi–Cox images

Dendrite segmentation was performed using a pix2pix conditional generative adversarial network (cGAN) machine learning model with a resolution of 256 × 256 pixels and three-color channels. The training dataset included annotated sections from four images with random patches and augmentations applied during training. Model weights were saved every 50 steps to mitigate memory loss, and the best model was selected post-training. During inference, the model was applied multiple times across the image with different offsets to avoid artifacts. The segmentation accuracy was assessed qualitatively by experts on 32 images. Post-inference processing to quantify dendrite properties was performed using custom Python code and relevant libraries. These metrics were then analyzed for correlations with aged *Mitf* ^*mi-vga9/mi-vga9*^ compared to C57BL/6J.

### Statistical analysis

At least three mice per genotype and age group were included in the study. Quantitative results were analyzed using two-way ANOVA and two-tailed unpaired Student’s *t*-tests using the R statistical package. To obtain *p*-values for ANOVA tests, multiple comparisons were conducted with Sidak’s and Tukey’s corrections. The numerical results represent the mean and standard deviation (SD). The criteria for significance levels used to generate volcano plots and bar graphs from RNA-seq analysis are described both in *Materials and Methods* and in the corresponding figure legends.

## Results

### Reduced olfactory ability is frequently observed in aged *Mitf* ^*mi-vga9/mi-vga9*^ mice

**FIGURE 1:**
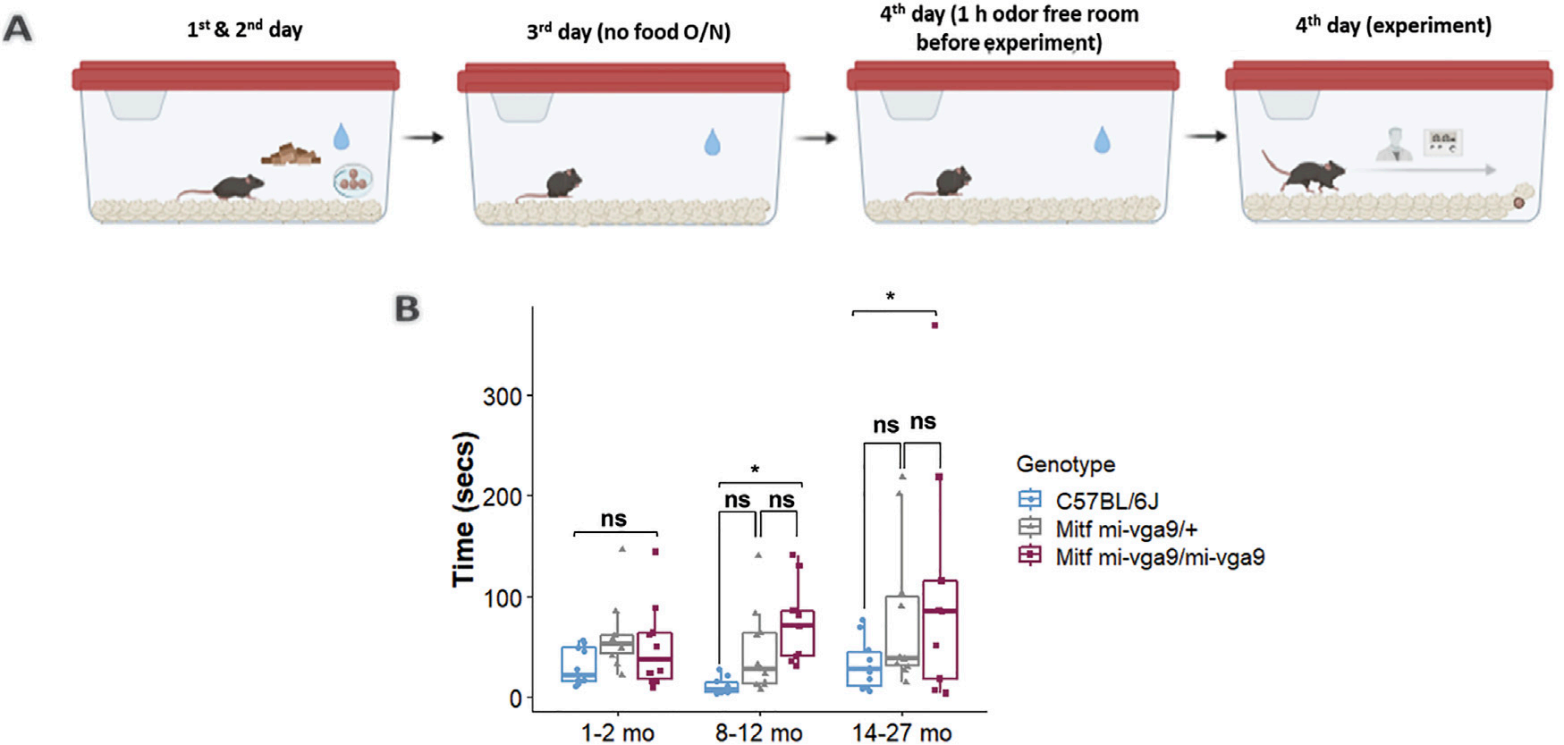
Aged *Mitf* ^*mi-vga9/mi-vga9*^ mice have reduced olfactory ability. **(A)** Setup of the hidden food assay. **(B)** Results of the hidden food assay showing the time spent by three age groups of C57BL/6J, *Mitf* ^*mi-vga9/+*^, and *Mitf* ^*mi-vga9/mi-vga9*^ mice to find the hidden cereal. N = 10 per genotype in age groups 1–2 months and 8–12 months; n = 10 for C57BL/6J and *Mitf* ^*mi-vga9/+*^, and n = 9 for 14–27-month-old *Mitf* ^*mi-vga9/mi-vga9*^. *p*-values were calculated using two-way ANOVA and adjusted with Tukey’s correction method. *p < 0.05; ns, not significant; mo, months old.

To assess the role of aging in the olfactory ability of *Mitf* ^*mi-vga9/mi-vga9*^ mice, a hidden food assay was performed with young and aged C57BL/6J, *Mitf* ^*mi-vga9/+*^, and *Mitf* ^*mi-vga9/mi-vga9*^ mice. The mice were divided into three age groups: 1–2, 8–12, and 14–27 months old. Diagrammatic representation of the assay setup is given in Figure 1A. Young *Mitf* ^*mi-vga9/mi-vga9*^ mice behaved similar to young C57BL/6J and *Mitf* ^*mi-vga9/+*^ mice, whereas aged *Mitf* ^*mi-vga9/mi-vga9*^ mice often had significantly reduced olfactory ability compared to aged C57BL/6J mice. The olfactory ability of 8–12-month-old *Mitf* ^*mi-vga9/mi-vga9*^ mice was 15% lower than that of C57BL/6J mice (8–12 months old: *t*_*(80)*_ *=−2.584*, *p = 0.030*8). In the age range of 14–27 months, *Mitf* ^*mi-vga9/mi-vga9*^ mice showed a 34.5% decrease in the olfactory ability compared to C57BL/6J mice (14–27 months old: *t*_*(80)*_ *=−2.969*, *p = 0.0109,* Tukey’s correction method) (Figure 1B). Variation in the time that *Mitf* ^*mi-vga9/+*^ and *Mitf* ^*mi-vga9/mi-vga9*^ mice spent searching was high, some showing a similar olfactory ability to C57BL/6J mice. In the aged groups, there were individual *Mitf* ^*mi-vga9/mi-vga9*^ mice that showed severely reduced olfactory ability. Interestingly, *Mitf* ^*mi-vga9/+*^ mice, which are phenotypically similar to C57BL/6J (i.e., black coat color, normal eyes, and presence of melanocytes and mast cells), showed a trend of reduced olfactory ability. This suggests that the effect is primary, rather than secondary due to *Mitf* pleiotropy and indicates a dosage effect associated with the *Mitf* mutation, which is in accordance with previous studies (Atacho et al., 2020; Ingason et al., 2019; Sabaté San José and Petersen, 2024).

### Potassium channel expression in the aged *Mitf* ^*mi-vga9/mi-vga9*^ OB

**FIGURE 2:**
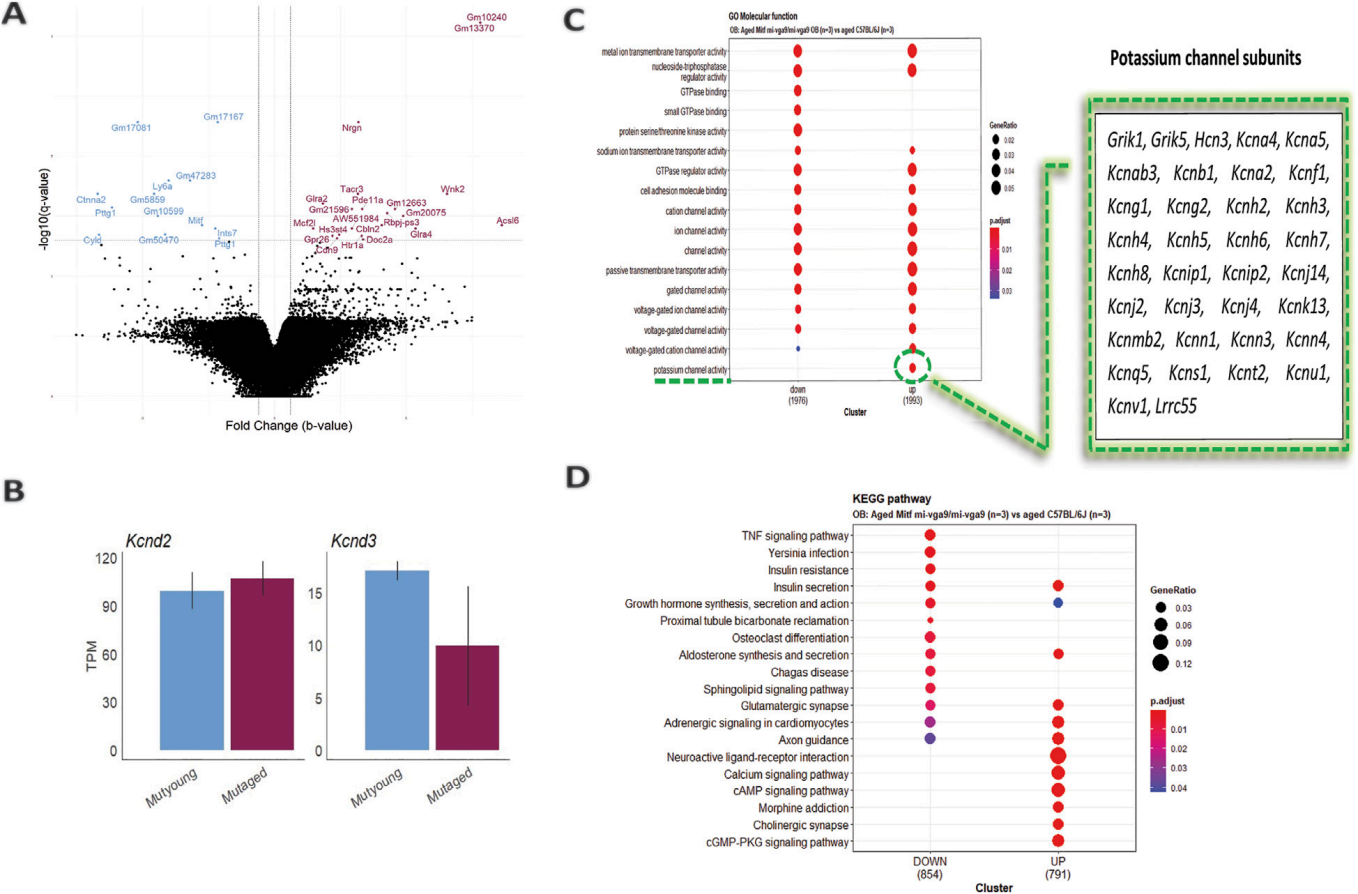
Increased potassium channel activity in the aged *Mitf* ^*mi-vga9/mi-vga9*^ OB. **(A)** Volcano plot shows DE genes in the OB of aged *Mitf* ^*mi-vga9/mi-vga9*^ mice compared to aged C57BL/6J mice. Blue indicates decreased DE genes, and purple indicates increased DE genes. N = 3 per genotype. **(B)** RNA expression of *Kcnd3* and *Kcnd2* in TPM in young (MUTyoung) and aged (MUTaged) *Mitf* ^*mi-vga9/mi-vga9*^ mice. N = 3 per age group. **(C)** GO Molecular Function (MF) analysis between aged *Mitf* ^*mi-vga9/mi-vga9*^ and aged C57BL/6J mice. The table shows genes enriched in the potassium channel activity cluster. **(D)** KEGG pathway analysis between aged *Mitf* ^*mi-vga9/mi-vga9*^ and aged C57BL/6J mice. N = 3 per genotype.

Loss of *Mitf* leads to increased neuronal activity in primary PNs of young *Mitf* ^*mi-vga9/mi-vga9*^ OBs due to reduced I_A_ potassium currents (Atacho et al., 2020). Single-molecule fluorescence *in situ* hybridization (smFISH) revealed decreased expression of potassium channel subunit *Kcnd3* (Kv4.3), correlated with increased neuronal activity in primary PNs of young *Mitf* ^*mi-vga9/mi-vga9*^ mice. On the other hand, the expression of *Kcnd2* (Kv4.2) was increased in M/T cells of *Mitf* ^*mi-vga9/mi-vga9*^ mice, possibly due to a compensatory mechanism (Atacho et al., 2020). Given the hyperactivity in young *Mitf* ^*mi-vga9/mi-vga9*^ OB primary M/T cells (Atacho et al., 2020), global gene expression analysis of aged *Mitf* ^*mi-vga9/mi-vga9*^ and C57BL/6J OBs was performed to examine the effects of aging on the expression of potassium channel subunits (Figure 2A; Supplementary Material provides a list of DE genes in the aged *Mitf* ^*mi-vga9/mi-vga9*^ OB). Aged mice in this and subsequent analysis were 14–27 months old. Indications of inflammation or degeneration were also of interest.

The potassium channel subunit (*Kcnd3*) previously shown to be reduced in expression in young *Mitf* ^*mi-vga9/mi-vga9*^ mice was not differentially expressed in aged *Mitf* ^*mi-vga9/mi-vga9*^ mice compared to aged C57BL/6J OB animals. It also remained similar when comparing aged and young *Mitf* ^*mi-vga9/mi-vga9*^ mice, suggesting that aging has no impact on global *Kcnd3* and *Kcnd2* expression (Figure 2B). A likely explanation is that the sensitivity and spatial resolution of smFISH allow this method to detect subtle changes in specific cell types, which the global RNA-seq misses.

To investigate the biological functions of differentially expressed genes between aged *Mitf* ^*mi-vga9/mi-vga9*^ and C57BL/6J OBs, Gene Ontology (GO) and Kyoto Encyclopedia of Genes and Genomes (KEGG) pathway analyses were performed. GO molecular function (GO MF) identified overlapping categories between genes with increased and decreased DE; the genes with decreased DE were enriched in GTPase binding, small GTPase binding, and protein serine/threonine kinase activity. However, in general, the expression of potassium channel subunits was increased in the aged *Mitf* ^*mi-vga9/mi-vga9*^ mice (Figure 2C). KEGG pathway analysis revealed that various pathways were enriched, and, again, there was considerable overlap between the up- and downregulated DE genes. Pathways such as TNF signaling, *Yersinia* infection, insulin resistance, proximal tubule bicarbonate reclamation, osteoclast differentiation, Chagas disease, and the sphingolipid signaling pathway were associated with the decreased DE genes. Pathways such as neuroactive ligand–receptor interaction, calcium signaling pathway, morphine addiction, cholinergic synapse, and the cGMP-PKG signaling pathway were enriched by the increased DE genes (Figure 2D). However, no clear functional connection was observed with neurodegeneration or inflammation.

### Dendritic morphology in OBs does not change upon aging of *Mitf* ^*mi-vga9/mi-vga9*^ mice

**FIGURE 3:**
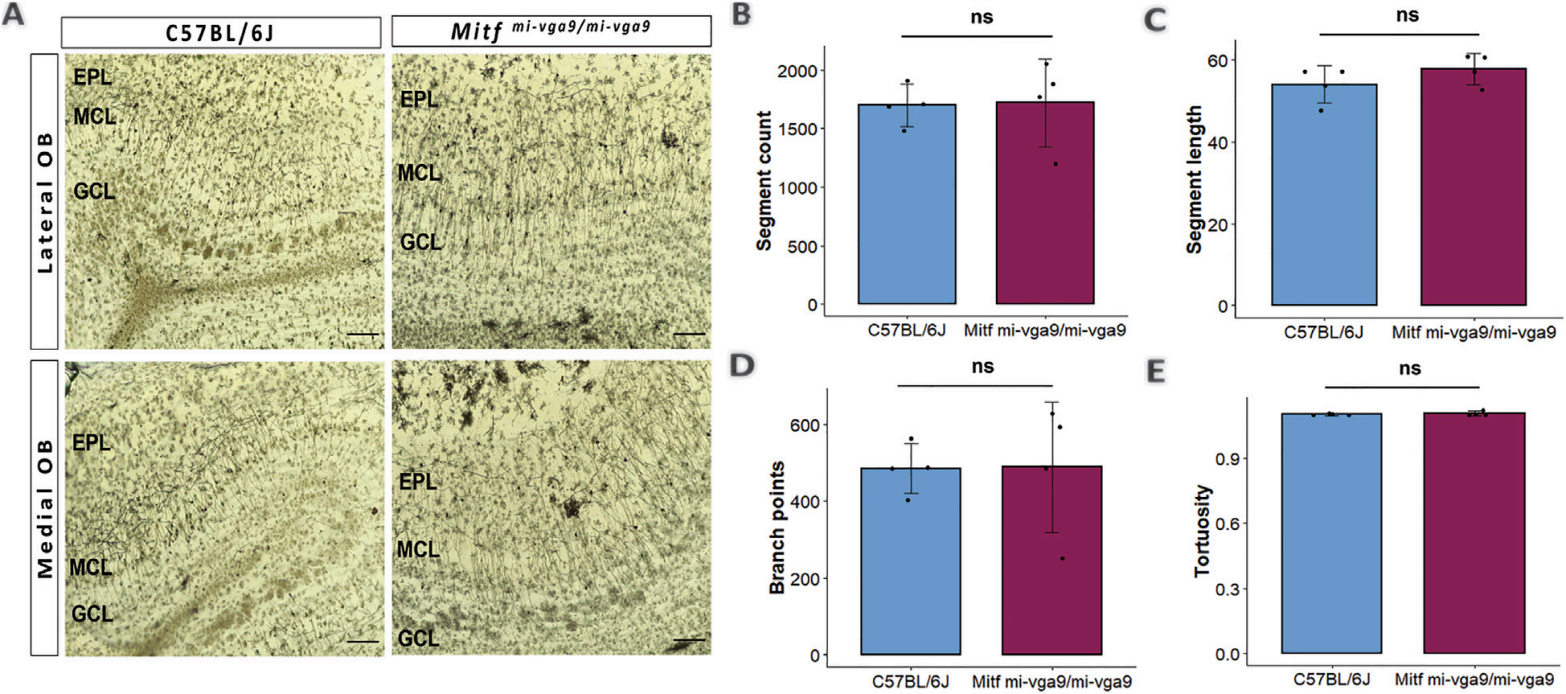
Dendritic morphology does not change upon aging of *Mitf* ^*mi-vga9/mi-vga9*^ OBs. **(A)** Representative Golgi–Cox images from the lateral and medial OBs of aged C57BL/6J and *Mitf* ^*mi-vga9/mi-vga9*^ mice. Analysis of Golgi–Cox images with regards to the number of segments **(B)**, segment length **(C)**, number of branch points **(D)**, and tortuosity **(E)**. Scale bar: 200 μm. N = 4 per genotype. Statistical analysis was performed using two-tailed unpaired Student’s *t*-test. EPL, external plexiform layer; MCL, mitral cell layer; GCL, granule cell layer; ns, not significant.

Dendritic morphology can be affected by long-term hyperactivity (Ugarte et al., 2023). To investigate this, the Golgi–Cox method was used to evaluate the morphology of the apical dendrites of M/T cells in aged C57BL/6J and *Mitf* ^*mi-vga9/mi-vga9*^ OBs (Figure 3A) (Vints et al., 2019; Zhong et al., 2019). Several parameters were quantified in each genotype, including the number of segments (Figure 3B), the segment length (Figure 3C), the number of branch points (Figure 3D), and tortuosity (Figure 3E). Analysis of these parameters revealed no differences between aged *Mitf* ^*mi-vga9/mi-vga9*^ and C57BL/6J OBs. Overall, the structure of dendrites was similar in both genotypes.

### Inflammatory status does not change in *Mitf* ^*mi-vga9/mi-vga9*^ OBs upon aging

*Mitf* has been shown to be an important regulator of the neurodegenerative and phagocytic transcriptional program of microglia (Dolan et al., 2023). Long-term hyperactivity in neurons is a frequent first step in neurodegeneration (Targa Dias Anastacio et al., 2022). Given the involvement of the MITF protein in the regulation of neuronal activity (Atacho et al., 2020), it is possible that the *Mitf* mutation may lead to neurodegeneration and subsequent inflammation, thereby contributing to the observed decrease in the olfactory ability.

**FIGURE 4:**
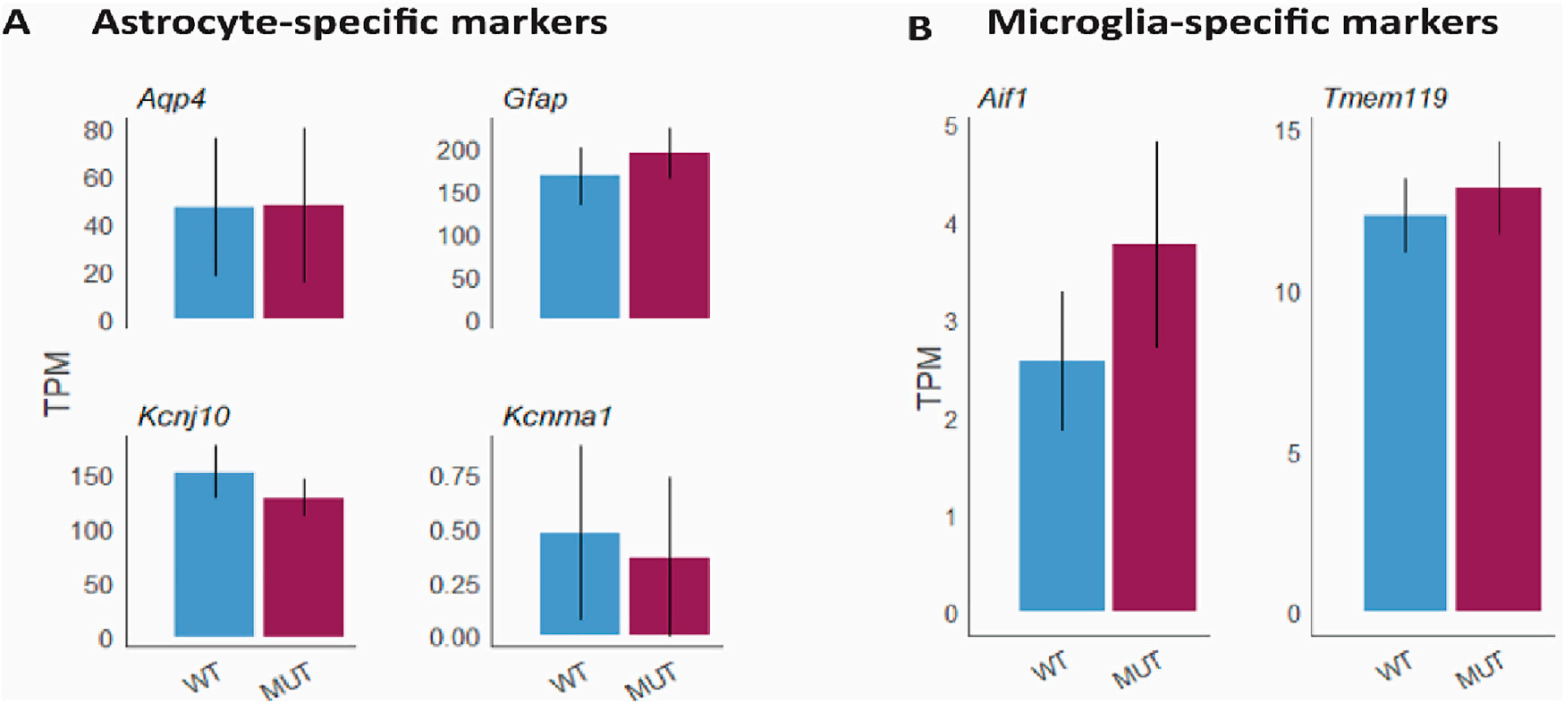
No change in the gene expression level of markers for astrocytes and microglia at the gene level in aged *Mitf* ^*mi-vga9/mi-vga9*^ OB. Expression of astrocyte-specific markers (*Aqp4, Gfap, Kcnj10,* and *Kcnma1*) **(A)** and microglia-specific markers (*Aif1,* aka *Iba-1*, and *Tmem119*) **(B)** in TPMs in the aged *Mitf* ^*mi-vga9/mi-vga9*^ (MUT) OB compared to the C57BL/6J (WT) OB. N = 3 per genotype.

To further analyze the potential effects of *Mitf* on neurodegeneration and inflammation during aging, the comparative gene expression analysis using RNA-seq was re-examined focusing on changes in genes associated to inflammation. The analysis showed that there were no changes in the expression of genes known to be specific to activated/reactive astrocytes (Figure 4A) or microglia activation (Figure 4B) between aged *Mitf* ^*mi-vga9/mi-vga9*^ and C57BL/6J OBs. This confirmed that the inflammatory status of *Mitf* ^*mi-vga9/mi-vga9*^ OBs remains unchanged upon aging.

### No evidence of neuroinflammation in the aged *Mitf* ^*mi-vga9/mi-vga9*^ OB

**FIGURE 5:**
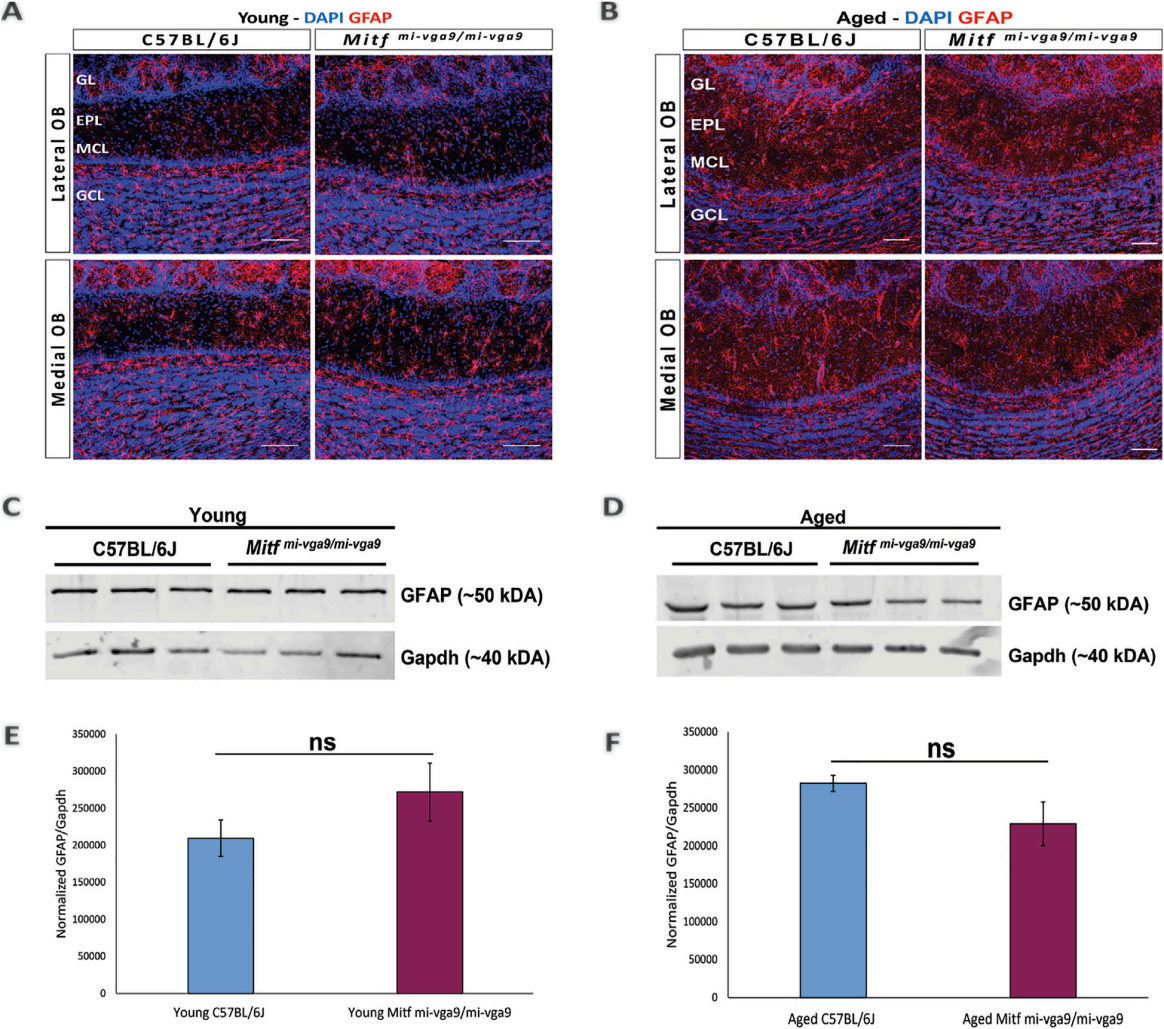
Expression of the astrocyte/astrogliosis marker (GFAP) is similar in young and aged *Mitf* ^*mi-vga9/mi-vga9*^ OBs. Representative immunofluorescence images show the expression of GFAP (red) in young **(A)** and aged **(B)** C57BL/6J and *Mitf* ^*mi-vga9/mi-vga9*^ OBs. N = 10 per genotype, and age group DAPI nuclear staining is shown in blue. Scale bar: 100 μm. Representative images of immunoblots from the lysate of young **(C)** and aged **(D)** C57BL/6J and *Mitf* ^*mi-vga9/mi-vga9*^ OBs probed for GFAP and Gapdh as a loading control. Densitometric analysis of GFAP bands normalized to Gapdh loading control in young **(E)** and aged **(F)** C57BL/6J and *Mitf* ^*mi-vga9/mi-vga9*^ mice. All immunoblotting experiments were performed with three mice (n = 3) per genotype and age group. Statistical analysis was performed using two-tailed unpaired Student’s *t*-test. GL, glomerular layer; EPL, external plexiform layer; MCL, mitral cell layer; GCL, granule cell layer; ns, not significant.

**FIGURE 6:**
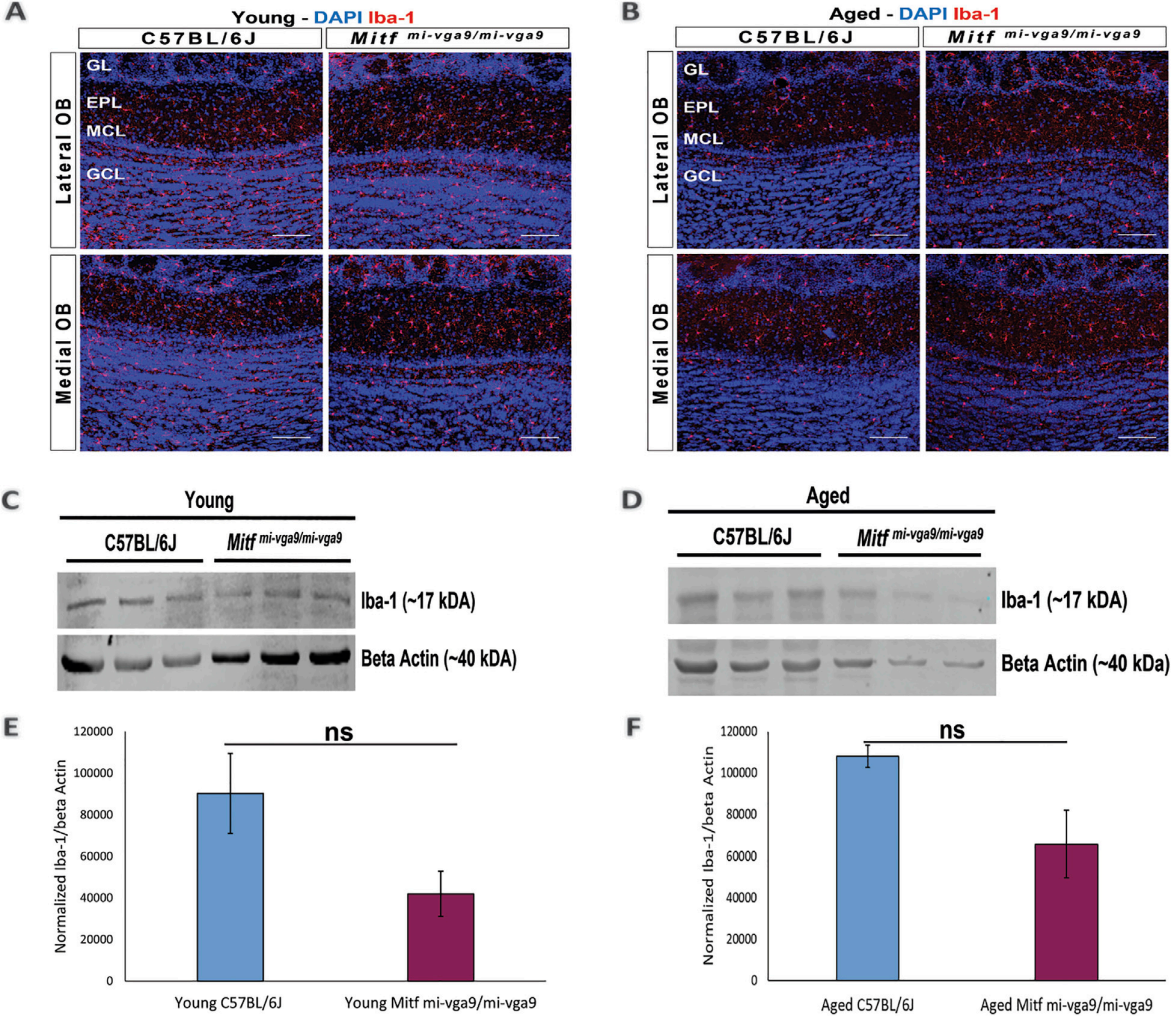
Expression of the microglia activation marker (Iba-1) does not change in young and aged *Mitf* ^*mi-vga9/mi-vga9*^ OBs. Representative immunofluorescence images for Iba-1 (red) in young **(A)** and aged **(B)** C57BL/6J and *Mitf* ^*mi-vga9/mi-vga9*^ OBs. N = 10 per genotype, and age group DAPI nuclear staining is shown in blue. Scale bar: 100 μm. Representative images of immunoblotting from the lysate of young **(C)** and aged **(D)** C67BL/6J and *Mitf* ^*mi-vga9/mi-vga9*^ OBs probed for Iba-1 and beta-actin as a loading control. Densitometric analysis of Iba-1 bands normalized to beta-actin in young **(E)** and aged **(F)** C57BL/6J and *Mitf* ^*mi-vga9/mi-vga9*^ OBs. All immunoblotting experiments were performed with three mice (n = 3) per genotype and age group. Statistical analysis was performed using two-tailed unpaired Student’s *t*-test. GL, glomerular layer; EPL, external plexiform layer; MCL, mitral cell layer; GCL, granule cell layer; ns, not significant.

To further examine inflammation at the protein level, immunohistochemistry was performed on OB sections of young and aged C57BL/6J and *Mitf* ^*mi-vga9/mi-vga9*^ mice using well-established neuroinflammation markers. Neuroinflammation and neurodegeneration in the CNS are generally studied by analyzing the state of astrocytes and microglia (Lucas et al., 2006) using GFAP, a marker for astrocytes and astrogliosis (Rodríguez et al., 2014), and Iba-1, a marker used to identify and characterize microglia (Streit et al., 2009). No pronounced changes were observed in the number of astrocytes in the OBs of young (Figure 5A) and aged (Figure 5B) C57BL/6J or *Mitf* ^*mi-vga9/mi-vga9*^ mice. These results were confirmed with Western blot analysis of GFAP, which showed no changes in the expression in young (Figures 5C, E) or aged (Figures 5D, F) *Mitf* ^*mi-vga9/mi-vga9*^ and C57BL/6J OBs. Likewise, there were no apparent changes in the number of microglia between OBs of young (Figure 6A) or aged (Figure 6B) *Mitf* ^*mi-vga9/mi-vga9*^ or C57BL/6J mice. Similar results were obtained in the Western blot analysis of Iba-1. No significant difference was observed in the expression of Iba-1 between young (Figures 6C, E) and aged (Figures 6D, F) *Mitf* ^*mi-vga9/mi-vga9*^ and C57BL/6J OBs. In summary, there was no evidence of neuroinflammation in aged *Mitf* ^*mi-vga9/mi-vga9*^ OBs based on the expression of glia markers.

### Quantification of PNs in the aging OB

**FIGURE 7:**
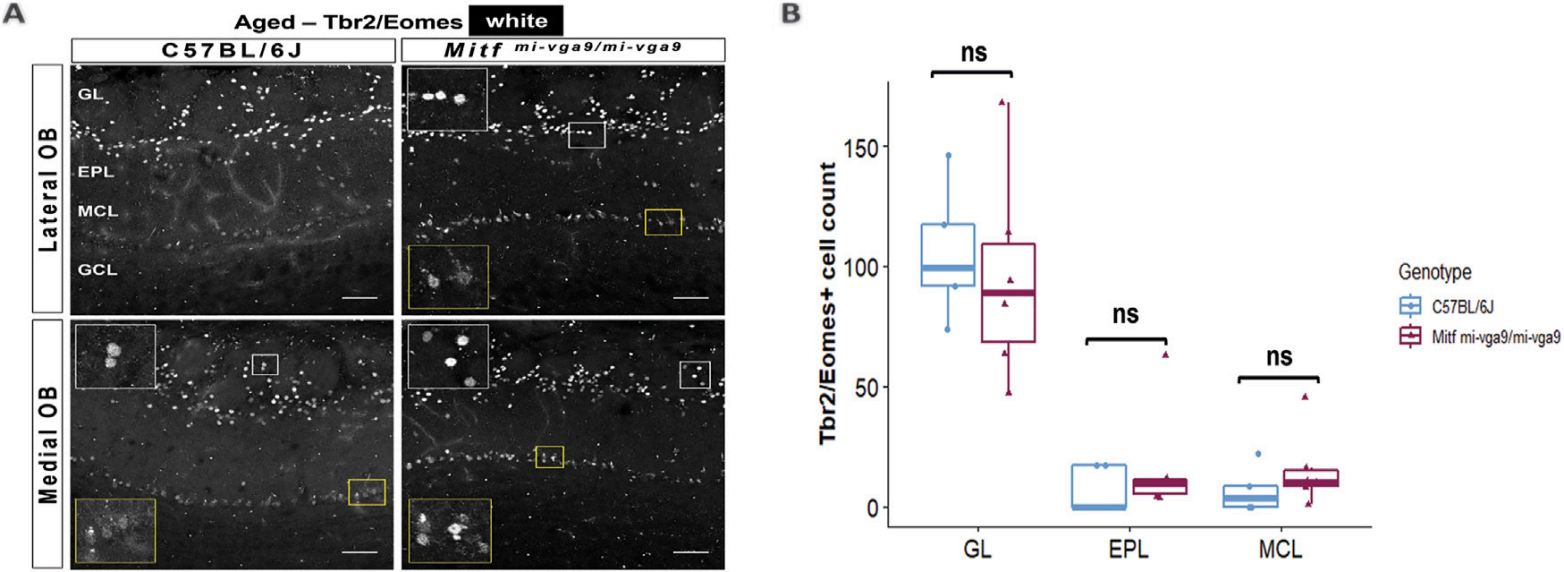
The number of Tbr2/Eomes-positive cells does not change upon aging. **(A)** Representative immunofluorescence images for Tbr2/Eomes (white) in aged C57BL/6J and *Mitf* ^*mi-vga9/mi-vga9*^ OBs. **(B)** Quantification of Tbr2/Eomes-positive cells in the GL, EPL, and MCL of aged C57BL/6J and *Mitf* ^*mi-vga9/mi-vga9*^ OBs. N = 5 for C57BL/6J and n = 6 for *Mitf* ^*mi-vga9/mi-vga9*^. Scale bar: 100 μm. Statistical analysis was performed using two-way ANOVA. GL, glomerular layer; EPL, external plexiform layer; MCL, mitral cell layer; GCL, granule cell layer; ns, not significant.

**FIGURE 8:**
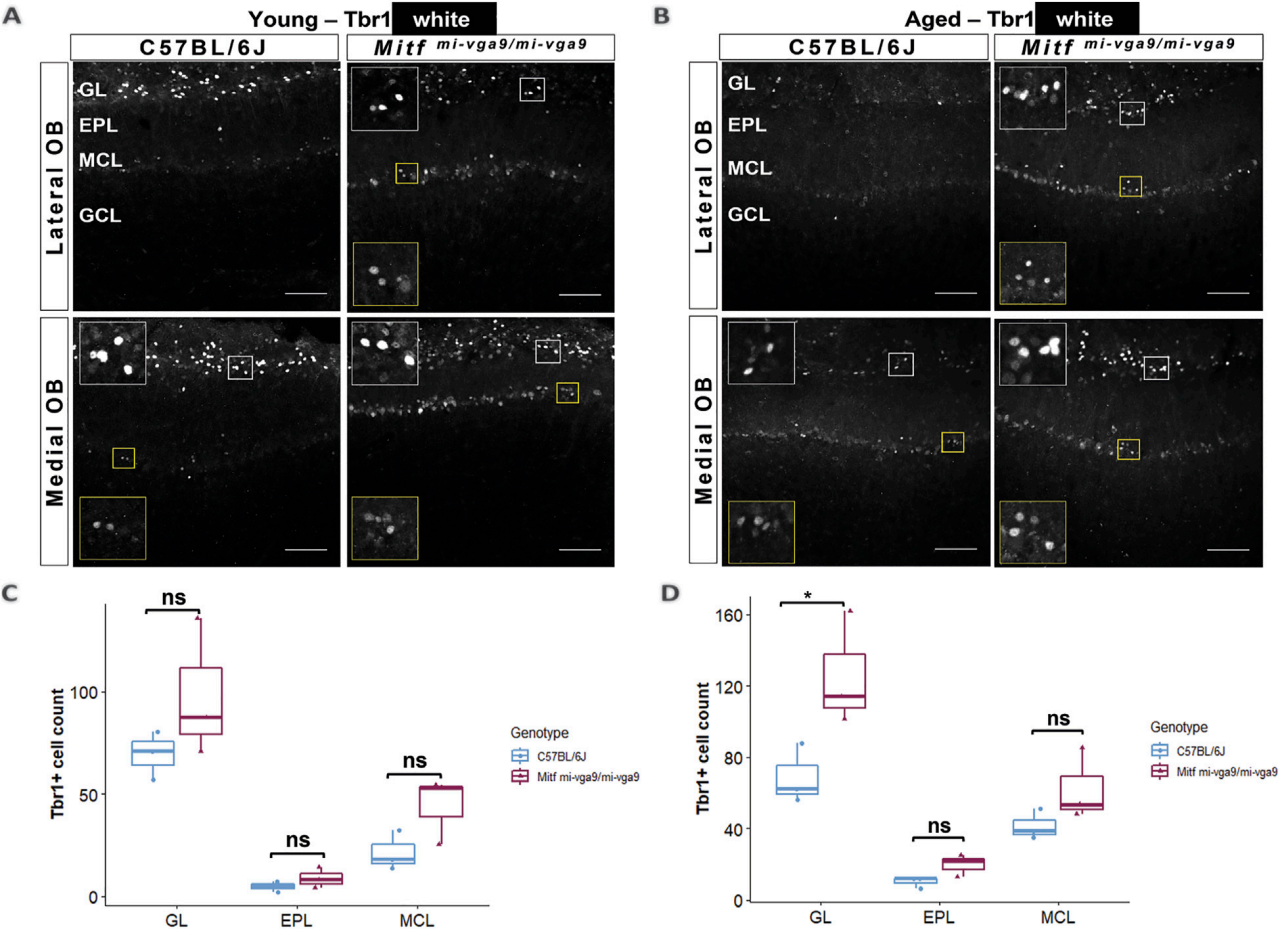
The number of Tbr1-positive cells is increased in the GL of aged *Mitf* ^*mi-vga9/mi-vga9*^ OBs. Representative immunofluorescence images for Tbr1 (white) in young **(A)** and aged **(B)** C57BL/6J and *Mitf* ^*mi-vga9/mi-vga9*^ OBs. Quantification of Tbr1-positive cells in the GL, EPL, and MCL of young **(C)** and aged **(D)** C57BL/6J and *Mitf* ^*mi-vga9/mi-vga9*^ OBs. N = 3 per genotype and age group. Scale bar: 100 μm. *p*-values were calculated using two-way ANOVA and adjusted with Sidak’s multiple comparison. *p < 0.05. GL, glomerular layer; EPL, external plexiform layer; MCL, mitral cell layer; GCL, granule cell layer; ns, not significant.

Neurodegeneration can result in the loss of neurons (Yankner et al., 2008). To investigate this in aged mice, we focused on the cells that normally express *Mitf* in C57BL/6J mice. OB tissues from young and aged C57BL/6J and *Mitf* ^*mi-vga9/mi-vga9*^ mice were stained with antibodies against two members of the Tbr1 subfamily of T-box transcription factors: Tbr2/Eomes and Tbr1. These proteins are expressed in glutamatergic neurons of the OB in a distinct and overlapping manner (Brill et al., 2009; Mizuguchi et al., 2012) (Figures 7, 8). Previous findings showed an increase in Tbr2/Eomes-positive cells in the GL, but not in the EPL or MCL of young *Mitf* ^*mi-vga9/mi-vga9*^ OBs (Atacho et al., 2020). However, the number of Tbr2/Eomes-positive cells was not different in GL, EPL, or MCL of aged *Mitf* ^*mi-vga9/mi-vga9*^ OBs compared to aged C57BL/6J OBs in the current study (Figure 7B). On the other hand, Tbr1 staining demonstrated that the number of Tbr1-positive cells was significantly increased in the GL in aged *Mitf* ^*mi-vga9/mi-vga9*^ OBs, whereas no change was observed in the EPL and MCL (Figure 8D; *t*_(12)_ =−3.987, *p* = 0.0267, Sidak’s multiple comparison). Similar trends can be seen in both young and aged mice. Taken together, while there are subtle changes in the composition and a possible increase in the subpopulations of glutamatergic neurons in the *Mitf* mutant OB, there was no reduction in the number of PNs with aging, arguing strongly against neuronal degeneration.

## Discussion

Reduction in olfactory ability, neuroinflammation, and neuronal hyperactivity often follow or precede neurodegeneration, e.g., in AD (Doty, 2012; Fullard et al., 2017; Murphy, 2019; Patino et al., 2021; Peters et al., 2003). Aging *Mitf* ^*mi-vga9/mi-vga9*^ mice often have reduced olfactory ability, and primary PNs from young *Mitf* ^*mi-vga9/mi-vga9*^ OB are hyperactive (Atacho et al., 2020). This could lead to increased neuronal or neural circuit stress *in vivo* and subsequently to neurodegeneration. The effects of *Mitf* are pleiotropic, and the loss of *Mitf* could also lead to degeneration through other mechanisms (Dolan et al., 2023), resulting in the reduction of olfactory ability.

The current study shows that aged *Mitf* ^*mi-vga9/mi-vga9*^ OBs have reduced olfactory ability with no clear evidence of neuroinflammation or neurodegeneration. The analysis was both on global signatures of neurodegeneration and on cell-autonomous effects on PNs, the key neurons expressing MITF. No increase in inflammation was detected upon aging of *Mitf* ^*mi-vga9/mi-vga9*^ mice, neither at the gene level nor in the expression of inflammatory proteins. Similarly, no reduction was observed in the number of OB PNs in *Mitf* ^*mi-vga9/mi-vga9*^ mice with aging. The increase in Tbr1-positive neurons in the *Mitf* ^*mi-vga9/mi-vga9*^ OB could reflect a reduction in the numbers of neurons found in a subgroup of tufted neurons, resulting in changes in the composition of glutamatergic neurons in the GL. Comparing gene expressions between aged C57BL/6J and aged *Mitf* ^*mi-vga9/mi-vga9*^ mice showed a high number of DE genes, with diverse functions and connected to diverse pathways. However, as the loss of *Mitf* leads to changes in the expression of two genes coding for potassium channel subunits in M/T cells, it was particularly interesting that there was a global increase in the expression of genes coding for potassium channel subunits during aging. Overall, such a change should lead to reduced neuronal activity. This suggests that with aging, neuronal hyperactivity leads to a reduction in neuronal activity. As complex mechanisms regulate the conduction of information in the OB, these changes are likely to be multifaceted (Burton, 2017), involving complicated interactions between various inhibitory neurons and neurotransmitter systems, contributing to the delicate balance of excitation and inhibition necessary for proper sensory processing (Abraham et al., 2010; Egger et al., 2003; Geramita and Urban, 2017). The disruption of this equilibrium could underlie the observed decrease in olfactory function (Gire et al., 2019) in aged *Mitf* ^*mi-vga9/mi-vga9*^ mice. Other possibilities could be a decrease in the function of the OE or OSNs. The *Mitf* mutation may also elicit effects potentially impacting processes such as proteostasis. MITF has been associated with autophagy in other cell types, including in melanoma cells (Möller et al., 2019). However, no specific changes were observed in the expression of genes that may explain such effects.

A caveat to this study is secondary effects due to the pleiotropy of *Mitf*. That the olfactory phenotype is also present in the heterozygous *Mitf* ^*mi-vga9*^ animals, phenotypically similar to C57BL/6J mice, supports this to be a primary effect. It can be concluded from the current study that the absence of *Mitf* does not lead to neurodegeneration with aging. However, *Mitf* could play a role in neurodegeneration induced by neuropathology, as in AD (Dolan et al., 2023). A direct challenge in a conditional model may unravel this.

## Data Availability

Gene expression data analyzed in this study is available in the GEO data repository under the accession number GSE277734. The raw data supporting the conclusions of this article will be made available by the authors, without undue reservation.
